# Association between sudden infant death syndrome and diphtheria-tetanus-pertussis immunisation: an ecological study

**DOI:** 10.1186/s12887-015-0318-7

**Published:** 2015-01-28

**Authors:** Jacqueline Müller-Nordhorn, Chih-Mei Hettler-Chen, Thomas Keil, Rebecca Muckelbauer

**Affiliations:** Berlin School of Public Health, Charité – Universitätsmedizin Berlin, Seestr. 73, 13347 Berlin, Germany; Institute for Social Medicine, Epidemiology and Health Economics, Charité – Universitätsmedizin Berlin, Luisenstr. 57, 10117 Berlin, Germany

**Keywords:** Sudden infant death syndrome, Diphtheria-tetanus-pertussis immunisation, Time trends

## Abstract

**Background:**

Sudden infant death syndrome (SIDS) continues to be one of the main causes of infant mortality in the United States. The objective of this study was to analyse the association between diphtheria-tetanus-pertussis (DTP) immunisation and SIDS over time.

**Methods:**

The Centers for Disease Control and Prevention provided the number of cases of SIDS and live births per year (1968–2009), allowing the calculation of SIDS mortality rates. Immunisation coverage was based on (1) the United States Immunization Survey (1968–1985), (2) the National Health Interview Survey (1991–1993), and (3) the National Immunization Survey (1994–2009). We used sleep position data from the National Infant Sleep Position Survey. To determine the time points at which significant changes occurred and to estimate the annual percentage change in mortality rates, we performed joinpoint regression analyses. We fitted a Poisson regression model to determine the association between SIDS mortality rates and DTP immunisation coverage (1975–2009).

**Results:**

SIDS mortality rates increased significantly from 1968 to 1971 (+27% annually), from 1971 to 1974 (+47%), and from 1974 to 1979 (+3%). They decreased from 1979 to 1991 (−1%) and from 1991 to 2001 (−8%). After 2001, mortality rates remained constant. DTP immunisation coverage was inversely associated with SIDS mortality rates. We observed an incidence rate ratio of 0.92 (95% confidence interval: 0.87 to 0.97) per 10% increase in DTP immunisation coverage after adjusting for infant sleep position.

**Conclusions:**

Increased DTP immunisation coverage is associated with decreased SIDS mortality. Current recommendations on timely DTP immunisation should be emphasised to prevent not only specific infectious diseases but also potentially SIDS.

## Background

Despite a major decrease in mortality since the early 1990s, sudden infant death syndrome (SIDS) continues to be one of the main causes of infant death worldwide [1]. In the United States, approximately 2,000 infants die from SIDS every year [2]. Many Western countries experienced a continuous increase in SIDS mortality during the 1970s - most likely due to a shift in diagnostic coding -, followed by a peak or plateau during the 1980s and a sharp decline at the beginning of the 1990s [1,3-5]. The decline in SIDS cases during the 1990s has been largely attributed to the ‘Back to Sleep’ campaigns, which promoted a non-prone sleep position [6]. It is often assumed that no other relevant changes affected the risk of SIDS during that time period [6].

However, one factor that also changed was the prevalence of pertussis immunisation. During the 1970s and 1980s, reports of neurological complications led to a dramatic decrease in immunisation coverage in many countries [7,8]. In the United States, immunisation coverage fell from 75% in 1975 to 64% in 1985 [9]. Finally, in 1991, the Institute of Medicine published a report that showed no increase in neurological complications associated with pertussis immunisation [10]. Pertussis immunisation, which is typically given in combination with diphtheria and tetanus vaccines (diphtheria-pertussis-tetanus, or DTP), thus quickly recovered beginning in 1991 [9].

Two meta-analyses of observational studies suggested that immunisation, and particularly DTP and oral polio vaccine (OPV), was associated with a significant reduction in the risk of SIDS [11,12]. It is unclear, however, whether the findings of these meta-analyses translate into recommendations to the public. Whereas some professional guidelines and recommendations have included immunisation as a measure for preventing SIDS, others have not [13,14]. In the general population, the fear of an increased risk of SIDS due to immunisation continues to exist [15]. This fear, in addition to other concerns about autism or vaccines ‘overloading’ the immune system, may lead parents to postpone immunisation during the first months of life or to refuse it altogether. Among infants younger than 5 months of age, only 80% receive the 2- and 4-month pertussis vaccines timely in the United States [16]. The objective of the current study was to analyse the ecological association between DTP immunisation coverage and SIDS incidence in the United States over several decades. We also describe trends in SIDS mortality, using joinpoint analyses to determine the time points at which significant changes occurred.

## Methods

### Mortality rates

We analysed trends in mortality rates of SIDS and related diagnostic groups between 1968 and 2009 in the United States. We used the following International Classification of Diseases (ICD) systems: the 8th revision (ICD-8) for the years 1968–1978, ICD-9 for 1979–1998, and ICD-10 for 1999–2009 (Table 1) [17]. The Centers for Disease Control and Prevention (CDC) provided the annual numbers of infant deaths and live births. Infant deaths were defined as deaths of infants less than 1 year of age. We calculated mortality rates by dividing the number of infant deaths by the number of live births. After the introduction of the SIDS diagnosis in 1969, SIDS mortality increased [18,19]. This increase seems to have been partially caused by a diagnostic shift to SIDS from other diagnoses, such as ill-defined causes of death, unintentional suffocation, and respiratory infections. We included related diagnostic groups to determine a cut-off after which an artefact due to coding seemed unlikely.

**Table 1:** **International Classification of Diseases (ICD) codes for sudden infant death syndrome and related diagnostic groups**

**Group of diagnoses**	**Individual diagnosis**	**ICD-8**	**ICD-9**	**ICD-10**
Symptoms, signs and ill-defined conditions		780–791, 793–796	780–799	R00–R99
	Sudden infant death syndrome	795	798.0	R95
	Ill-defined and unknown causes of mortality	795–796	798.1–799.9	R96–R99
	Other symptoms, signs and ill-defined conditions	793–794	780–797	R00–R94
Unintentional suffocation		E911–913*	E911–913*	W75–W84*
Diseases of the respiratory system		460–519	460–519	J00–J99

*includes diagnoses such as accidental suffocation and strangulation in bed (E913.0/W75) and unspecified threat to breathing (E913.9/W84).

### Immunisation coverage

We retrieved data on DTP, OPV, and *Haemophilus influenzae* type b (Hib) immunisation coverage using existing surveys [9,15,20,21]. DTP, OPV, and the Hib vaccine are all scheduled during the first 6 months of life (at 2, 4, and 6 months), which is the peak age range for SIDS [22]. To ensure comparability over time, we used the reported coverage of at least three doses of the respective vaccine. From 1995 onwards, any pertussis vaccine was reported including acellular pertussis vaccine (DTaP) [16].

The following three surveys provided data on immunisation coverage in the United States: the United States Immunization Survey (USIS; 1968–1985), the National Health Interview Survey (NHIS; 1991–1993), and the National Immunization Survey (NIS; 1994–2009) [9,15,20,21]. The USIS started as an area-probability household survey using face-to-face interviews and became a telephone survey in 1971 [9]. Until 1978, the USIS assessed the immunisation of children between 1 and 4 years of age. Between 1979 and 1985, the survey included only children aged 24–35 months. The collected information was based on either parental recall or an immunisation record that was maintained at home. There was a lack of information on immunisation coverage from 1986–1990. From 1991 onwards, the NHIS assessed the immunisation status of children aged 19–35 months [20,23,24]. The NHIS examined a representative probability sample of households in the United States using face-to-face interviews. If a child’s immunisation records were available, the data were abstracted from the records; otherwise, the collected information was based on parental recall. Then, in 1994, the CDC implemented the NIS for continuous monitoring of immunisation coverage [15,21]. For 1994, we used the Morbidity and Mortality Weekly Report, and for the years 1995–2009, we used the public use files that are published on the CDC website [15,16]. The NIS is a random-digit-dialling telephone survey of households with children aged 19–35 months. The data were validated with the immunisation history of the child, which was obtained from the family’s health care provider [25]. The NIS and NHIS yielded similar results for estimated immunisation coverage levels [15]. In the current study, immunisation coverage is presented graphically as the percentage of children who were immunised with DTP, OPV, and the Hib vaccine in each year during the time period from 1968 to 2009.

### Infant sleep position

The National Infant Sleep Position (NISP) Study began to assess the sleep positions of infants in the general population of the United States in 1992 [26,27]. Annual telephone surveys of randomly selected households with infants were conducted [26]. For the present analyses, we retrieved data on sleep position (prevalence of infants with a supine sleep position) until 2009 from the NISP Study [28]. We extended the first value backward in time from 1992, assuming that the 1992 value reflected the traditional way of putting infants to sleep prior to the ‘Back to Sleep’ campaign [5,28].

### Ethical approval

The ethics committee of the Charité – Universitätsmedizin Berlin approved the study.

### Statistical analyses

Joinpoint regression analyses, also called segmented or piecewise regression analyses, were conducted to identify the specific years (joinpoints) when significant changes in trends of mortality rates occurred. Joinpoint regression splits the data into segments and fits the trends for each segment. The annual percentage change (APC) in the mortality rates of SIDS and related diagnostic groups within all segments was estimated. The APC described the percentage change in the mortality rates in a specific year compared to the previous year. The analysis assumed that mortality rates changed at a constant percentage every year during the defined time periods between joinpoints. Log-linear models were fitted for mortality rates and the number of possible joinpoints was set between 0 and 5. The number of joinpoints was verified using a permutation test. For the annual percentage change estimates, 95% confidence intervals (CIs) were calculated. The Joinpoint Regression Program version 4.0.1 (National Cancer Institute, Calverton, MD, USA) was used.

Based on the results of the joinpoint analyses, the multivariable analysis of the association between SIDS mortality rates and DTP immunisation coverage was restricted to the years 1975–2009. The large increases in SIDS rates in the years 1968–1971 and 1971–1974 were most likely caused by changes in coding. As a consequence, the analysis was started after the point of change in the year 1974 which resulted in the analysed period of the years 1975–2009. First, lowess smoothers, a non-parametric regression method, were plotted to visualise the association between SIDS rates and DPT immunisation coverage. Then, multivariable generalised additive models were fitted using the procedure GAM of the statistical software package R (R Foundation, Vienna, Austria). GAM is an exploratory data analysis tool that evaluates the linearity of associations. We evaluated the association between SIDS mortality rates as the dependent variable and the independent variables of DTP immunisation coverage and prevalence of the supine sleep position. Because the dependent variable was mortality rate, the log-link function was used. The initial assessment showed that the associations between SIDS mortality rates and DTP immunisation coverage, adjusted for the prevalence of the supine sleep position, were linear. Therefore, a Poisson regression model was built as the final model for SIDS mortality rates using the procedure GLM in R with the log-link function. The independent variables of this model included DTP immunisation coverage and prevalence of the supine sleep position. We also considered OPV and Hib immunisation coverage as independent variables. Spearman’s rank correlation was used to determine the correlations among DTP, OPV, and Hib immunisation coverage. The correlation coefficients were 0.96 between DTP and OPV, and 0.65 between DTP and Hib immunisation coverage (from 1992 onward). Thus, the final Poisson regression model did not include OPV and Hib immunisation coverage due to collinearity. To investigate the association between OPV immunisation coverage and SIDS mortality rates, we built a separate Poisson regression model with OPV immunisation coverage and prevalence of the supine sleep position as the independent variables. The association between SIDS mortality rates and Hib immunisation coverage was not investigated because the corresponding data were not available before 1992. The estimates are presented as incidence rate ratios with 95% CIs. The analyses were performed using R version 2.15.1 with the additional MGCV package.

## Results

**Figure 1:**
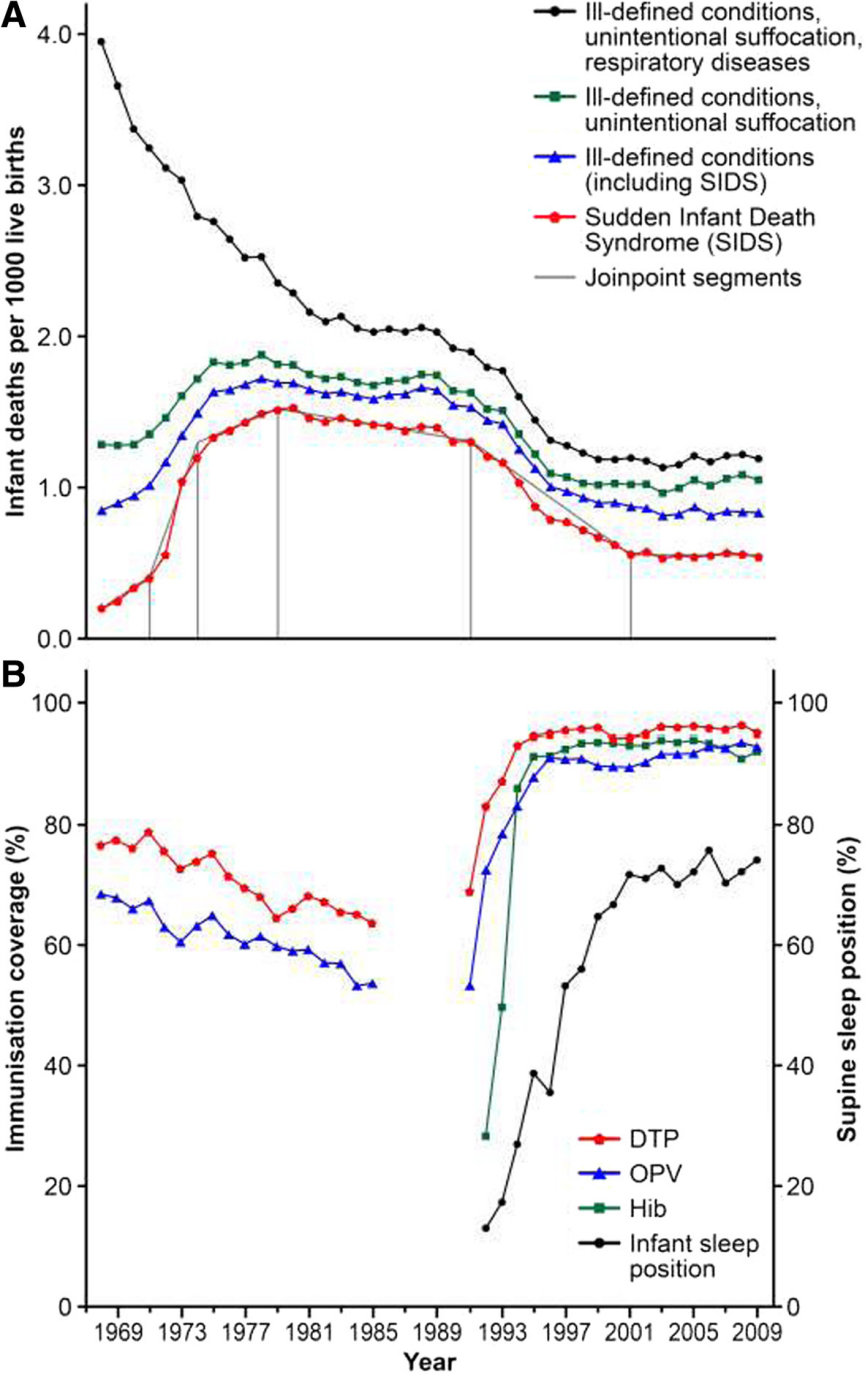
**Association between sudden infant death syndrome (SIDS) and immunisation coverage in the United States over time. A)** Trends in mortality rates of sudden infant death syndrome (SIDS) and related diagnostic groups in the United States over time. Joinpoints indicate years with significant changes in SIDS mortality. **B)** Trends in immunisation coverage and infant sleep positions in the United States over time; sources: United States Immunisation Survey 1968–1978 for children aged 1–4 years and 1979–1985 for children aged 24–35 months [9], National Health Interview Survey 1991–1993 for children aged 19–35 months [23,24], National Immunisation Survey 1994–2009 for children aged 19–35 months[15,16], and National Infant Sleep Position Study 1992–2009 [28]. Abbreviations: DTP, diphtheria-pertussis-tetanus (including any acellular pertussis from 1995 onwards) [16]; Hib, *Haemophilus influenzae* type b; OPV, oral polio vaccine

In the United States, trends in the annual SIDS mortality rates changed significantly in the years 1971, 1974, 1979, 1991, and 2001 (Figure 1, Table 2). Mortality rates increased from 1968 to 1971, from 1971 to 1974, and from 1974 to 1979. The APCs in mortality rates were 26.5% and 47.3% during the first two time periods, followed by a smaller increase of 3.3% until 1979. From 1979 to 1991, SIDS mortality rates began to decrease, with an APC of −1.2%. After 1991, mortality rates decreased to a larger extent, with an APC of −8.3% until 2001. After 2001, SIDS mortality rates remained constant. Similar trends were present in the related diagnostic groups, although the increases during the 1970s were less pronounced (Figure 1, Table 2).

**Table 2:** **Joinpoint analyses of trends in mortality rates of sudden infant death syndrome (SIDS) and related diagnostic groups**

**Diagnostic group**		**Time periods defined by joinpoint regression**					
SIDS	Period	1968–1971	1971–1974	1974–1979	1979–1991	1991–2001	2001–2009
	APC (95% CI)	26.5 (20.7, 32.5)*	47.3 (34.2, 61.7)*	3.3 (0.2, 6.3)*	−1.2 (−1.8, −0.6)*	−8.3 (−9.1, −7.5)*	−0.1 (−1.1, 1.0)
Symptoms, signs and ill-defined conditions	Period	1968–1971	1971–1975	1975–1991	1991–1998	1998–2009	
	APC (95% CI)	6.6 (2.7, 10.7)*	13.5 (9.3, 17.8)*	−0.4 (−0.7, −0.1)*	−7.8 (−9.0, −6.6)*	−0.8 (−1.3, −0.3)*	
Symptoms, signs and ill-defined conditions plus unintentional suffocation	Period	1968–1970	1970–1975	1975–1991	1991–1998	1998–2009	
	APC (95% CI)	−0.6 (−8.0, 7.5)	7.7 (5.1, 10.4)*	−0.7 (−1.0, −0.3)*	−7.0 (−8.2, −5.8)*	0.5 (−0.0, 1.0)	
Symptoms, signs and ill-defined conditions, unintentional suffocation plus respiratory diseases	Period	1968–1970	1970–1982	1982–1991	1991–1998	1998–2009	
	APC (95% CI)	−7.8 (−13.3, −1.9)^*^	−3.8 (−4.2, −3.4)^*^	−0.8 (−1.5, −0.2)*	−6.9 (−7.9, −5.9)^*^	0.1 (−0.4, 0.5)	

APC, annual percentage change (the number of periods was defined by the joinpoints that were derived from the best-fit models); CI, confidence interval.

^*^APC significantly different from 0 (P < 0.05).

Figure 1 shows time trends in mortality rates, immunisation coverage, and infant sleep position. The multivariable Poisson regression indicated that the risk of SIDS was inversely associated with DTP immunisation coverage between 1975 and 2009. The estimated incidence rate ratio for SIDS was 0.92 (95% CI: 0.87 to 0.97) for every 10% increase in DTP immunisation coverage, adjusted for the prevalence of the supine sleep position (Table 3). Similarly, the second regression model including OPV immunisation coverage indicated that the risk of SIDS was inversely associated with increasing OPV immunisation coverage, adjusted for the prevalence of the supine sleep position (Table 4).

**Table 3:** **Association between diphtheria-tetanus-pertussis (DTP) immunisation coverage and mortality rates of sudden infant death syndrome (SIDS) (United States, 1975–2009)**

	**Estimate [per 10% increase]**	**IRR (95% CI)**
DTP immunisation coverage	−0.08	0.92 (0.87, 0.97)
Infant sleep position (supine)	−0.12	0.89 (0.86, 0.91)

CI, confidence interval; IRR, incidence rate ratio.

**Table 4:** **Association between oral polio vaccine (OPV) immunisation coverage and mortality rates of sudden infant death syndrome (SIDS) (United States, 1975–2009)**

	**Estimate [per 10% increase]**	**IRR (95% CI)**
OPV immunisation coverage	−0.07	0.94 (0.89, 0.98)
Infant sleep position (supine)	−0.12	0.89 (0.86, 0.92)

CI, confidence interval; IRR, incidence rate ratio.

## Discussion

This study is the first ecological analysis of trends in SIDS mortality rates and their association with DTP immunisation coverage over a time period of nearly 40 years. SIDS mortality rates have been inversely associated with DTP immunisation coverage in the United States over recent decades. The major increases in SIDS rates from the late 1960s to 1974 as shown by the current study’s joinpoint analyses were most likely due to a shift in coding. After 1974, SIDS mortality rates stabilised with only minor increases until 1979. SIDS mortality rates started to decrease slightly between 1979 and 1991. The most notable decreases in SIDS rates occurred from 1991 onwards, coinciding with increases in DTP immunisation.

Meta-analyses have shown that DTP immunisation, with or without OPV or the Hib vaccine, is associated with a reduced risk of SIDS [11,12]. One of the meta-analyses included 9 case–control studies and showed a pooled multivariate odds ratio of 0.54 (95% CI: 0.39 to 0.76) [11]. The other meta-analysis included 4 case–control studies and 1 cohort study and had a pooled risk ratio of 0.67 (95% CI: 0.60 to 0.75) [12]. In 2011, the Task Force on Sudden Infant Death Syndrome included immunisation as one of the recommendations to reduce the risk of SIDS [13]. However, recommendations to the public and the ‘grey literaure’ often do not include immunisation in the prevention of SIDS. Prevailing safety concerns with regard to immunisation may have played a role in this hesistance for many years. Studies showing a reduction of SIDS associated with immunisation have typically reported their results cautiously, often using double negations [12,29]. For example, the National Institute of Child Health and Human Development SIDS Cooperative Epidemiological Study showed that the relative risk of SIDS associated with DTP immunisation was 0.54 in cases compared with controls (P < 0.001, no CI reported) [29]. The authors carefully concluded that “DTP immunisation was not a significant factor in the occurrence of SIDS” [29].

DTP immunisation may protect against SIDS by preventing infection with *Bordetella* (*B*.) *pertussis*. SIDS might thus be undiagnosed pertussis [30]. In pertussis, the initial symptoms resemble a non-specific, flu-like illness and persist for approximately 7 days [31]. Infants with pertussis may not develop typical symptoms such as paroxysmal coughing or a whoop, and diagnostic tests have low sensitivity during the early stages of the disease [31]. In approximately 50–80% of SIDS cases, signs of upper and lower respiratory tract infection, characterised by a mild cellular infiltrate, have been found [22]. Pertussis-associated gasping may induce internal upper airway obstruction, which is consistent with the intrathoracic petechiae typically found in SIDS cases [32]. However, a case–control study investigating the association between *B. pertussis* infection and SIDS did not show a difference in the prevalence of *B. pertussis* between the SIDS cases and the controls (5.1% vs. 5.3%, respectively) [33]. In the case of an association, SIDS might occur at a time when *B. pertussis* is not yet or no longer detectable. More studies are needed as evidence from only one case–control study – even if well-done - is too sparse to draw a conclusion.

*B. pertussis* infection may have indirect effects such as the impairment of the immune system and an increased likelihood of co-infections [31,34]. DTP immunisation may also induce cross-reactivity to other agents and their products [35]. These findings may indicate either a lack of sensitivity in diagnostic testing or a protective effect of the immunisation, independent of direct prevention of *B. pertussis* infection.

In the current study, DTP immunisation was highly correlated with OPV and Hib immunisation. Furthermore, similar to DTP immunisation, OPV immunisation was associated with a reduced risk of SIDS. Case–control studies have associated a similar reduction in SIDS risk with DTP and OPV immunisation, whereas less evidence is available regarding Hib immunisation [11,12,29]. In most countries, OPV immunisation did not decrease to the same extent as in the United States. In England, for example, OPV immunisation coverage remained at approximately 80%, with the trends in SIDS mortality being similar to the trends in the United States [4,36]. Hib immunisation was introduced in many countries during the early 1990s, and this vaccine may be a responsible agent in the prevention of SIDS as well [24,36]. In addition to the pertussis component, DTP includes diphtheria and tetanus components. Certain countries, such as England and Sweden, previously experienced major decreases in pertussis immunisation but administered diphtheria and tetanus vaccines separately, thus maintaining high coverage [36,37]. The SIDS trends in these countries were similar to the trends in the United States [4,5]. Thus, diphtheria and tetanus immunisation seem less likely to be associated with SIDS.

Changes in the coverage of pertussis immunisation, the recommended schedule, and the type of immunisation often coincided with the promotion of the non-prone sleep position for infants [4,6,9,36]. This phenomenon renders the disentangling of individual effects on SIDS mortality difficult. The increasing prevalence of the supine sleep position during the 1990s coincided with the increase in DTP immunisation coverage in the United States. Similarly, in England, the national ‘Back to Sleep’ campaign and the resumption of pertussis immunisation of the population both occurred during the early 1990s [4,36]. In West Germany, for example, pertussis immunisation was removed from the national recommendations between 1974 and 1991 [8]. The reintroduction of pertussis immunisations in 1991 occurred at the same time as regional campaigns promoting the non-prone sleep position [3]. The lack of standardised assessments of both pertussis immunisation coverage and infants’ sleep positions, however, hinders regional comparisons over time.

The current study has several limitations. A major limitation is the use of historic data and the lack of uniform assessment of pertussis immunisation. Three different surveys provided the data that were used in the study [9,20,21]. In particular, the USIS had several known methodological weaknesses that led to its discontinuation in 1985 [9]. These weaknesses included the shift from using a household-based survey to a telephone-based survey in 1971 and the mixed use of parental recall and immunisation records. The NHIS survey also changed its methodology between 1991 and 1992 [23]. The third survey, the NIS, was a telephone-based survey [21]. Despite adjustments, differences in immunisation coverage between households with and without a telephone cannot be excluded. An additional limitation is the lack of data on immunisation for the years 1986–1990. Data on sleep position were only available beginning in 1992 [28]. Carrying the first value (13%, supine sleep position) backward might have underestimated the association between SIDS mortality and sleep position if the supine sleep position had been more prevalent during the first years of the analysis.

Finally, ecological studies are at a higher risk of being affected by confounders compared with other epidemiological studies due to the use of aggregated data. Both immunisation and the supine sleep position were more prevalent in populations with a higher socioeconomic status during the early 1990s [23,24,27]. Socioeconomic status is linked to a number of other health-related behaviours that potentially affect the occurrence of SIDS [38]. Potential confounders at the population level include changes in the prevalence of smoking, breastfeeding, and pacifier use, as well as changes in sleep environment and socioeconomic conditions, including cultural background [39-41].

## Conclusion

DTP immunisation is inversely associated with SIDS mortality on the population level. The current findings may strengthen parents’ confidence in the benefit of DTP immunisation, especially as they are supported by the results of two meta-analyses [11,12]. As a public health measure, it is important to emphasise the need for timely immunisation in accordance to the existing schedule. Although confounding and the ecological fallacy due to the use of aggregate data cannot be excluded further research on potential underlying mechanisms of the association between SIDS and immunisation is warranted.

### Availability of supporting data

Only public use files and published data were analysed. References to the data sources are indicated in the text.

## Abbreviations

APC: Annual percentage change
B. pertussis: Bordetella pertussis
DTP: diphtheria-tetanus-pertussis
DTaP: diphtheria-tetanus-acellular pertussis
NHIS: National Health Interview Survey
NIS: National Immunization Survey
NISP: National Infant Sleep Position
OPV: oral polio vaccine
SIDS: sudden infant death syndrome
USIS: United States Immunization Survey
